# Neonatal Isoflurane Exposure Induces Neurocognitive Impairment and Abnormal Hippocampal Histone Acetylation in Mice

**DOI:** 10.1371/journal.pone.0125815

**Published:** 2015-04-30

**Authors:** Tao Zhong, Qulian Guo, Wangyuan Zou, Xiaoyan Zhu, Zongbin Song, Bei Sun, Xin He, Yong Yang

**Affiliations:** Department of Anesthesiology, Xiangya Hospital of Central South University, Changsha, Hunan Province, PR China; Radboud University, NETHERLANDS

## Abstract

**Background:**

Neonatal exposure to isoflurane may induce long-term memory impairment in mice. Histone acetylation is an important form of chromatin modification that regulates the transcription of genes required for memory formation. This study investigated whether neonatal isoflurane exposure-induced neurocognitive impairment is related to dysregulated histone acetylation in the hippocampus and whether it can be attenuated by the histone deacetylase (HDAC) inhibitor trichostatin A (TSA).

**Methods:**

C57BL/6 mice were exposed to 0.75% isoflurane three times (each for 4 h) at postnatal days 7, 8, and 9. Contextual fear conditioning (CFC) was tested at 3 months after anesthesia administration. TSA was intraperitoneally injected 2 h before CFC training. Hippocampal histone acetylation levels were analyzed following CFC training. Levels of the neuronal activation and synaptic plasticity marker c-Fos were investigated at the same time point.

**Results:**

Mice that were neonatally exposed to isoflurane showed significant memory impairment on CFC testing. These mice also exhibited dysregulated hippocampal H4K12 acetylation and decreased c-Fos expression following CFC training. TSA attenuated isoflurane-induced memory impairment and simultaneously increased histone acetylation and c-Fos levels in the hippocampal cornu ammonis (CA)1 area 1 h after CFC training.

**Conclusions:**

Memory impairment induced by repeated neonatal exposure to isoflurane is associated with dysregulated histone H4K12 acetylation in the hippocampus, which probably affects downstream c-Fos gene expression following CFC training. The HDAC inhibitor TSA successfully rescued impaired contextual fear memory, presumably by promoting histone acetylation and histone acetylation-mediated gene expression.

## Introduction

The use of inhaled anesthetics has become widespread in the pediatric population, and its deleterious effects are causing increasing concern. Several recent studies showed that children with multiple exposures to anesthesia and surgery before 4 years of age could be at increased risk of developing learning disabilities[1, 2]. Rodent studies also indicated that inhaled anesthetics differentially affect cognitive function in various developmental periods, and the developing brain of the neonatal animal seems to be particularly vulnerable to anesthetic-induced neurotoxicity [3, 4]. Isoflurane is a traditional inhaled anesthetic and has been demonstrated to induce more apoptotic neurodegeneration than sevoflurane [5]. Although a growing number of studies have focused on anesthetic-induced neurocognitive impairment, there are few effective interventions to prevent and treat such deleterious effects.

The capabilities to form and retrieve long-term memories are regarded as major aspects of cognitive function. Generally, changes in gene expression immediately following learning are thought to be indispensable for long-term memory formation. A wide variety of mechanisms regulate gene expression, and among chromatin remodeling via histone acetylation plays a particularly important role. Recent studies have demonstrated that cognitive function is closely related to histone acetylation alterations in the central nervous system, and dysregulation of hippocampal histone acetylation has particular significance for neurocognitive impairment associated with mutations, brain aging, iron overload, and other precipitating factors[6–8].

Histone acetyltransferases (HATs) catalyze histone acetylation, whereas histone deacetylases (HDACs) have the opposite effect. In previous studies, HDAC inhibitors (HDACi) such as sodium butyrate or trichostatin A (TSA) were reported to rescue memory deficits in both aged and gene-mutant mice by elevating the level of hippocampal histone acetylation, and these compounds also showed therapeutic potential for depression and some neurodegenerative disorders such as Huntington’s disease (HD), Parkinson's disease (PD), and Alzheimer’s disease (AD) [6, 8–13].

We therefore hypothesized that dysregulation of histone acetylation was involved in neurocognitive impairment caused by repeated neonatal exposure to isoflurane and that cognition impairment could therefore be ameliorated by the HDACi TSA. To test this hypothesis, we treated mice with 0.75% isoflurane for 4 h on postnatal days 7, 8, and 9 and assessed hippocampal histone acetylation and neurocognitive function using contextual fear conditioning (CFC) testing at 3 months after isoflurane exposure. Together with CFC, we carried out an open-field analysis to assess locomotor activity and anxiety levels in mice. In addition, we also determined whether TSA reversed changes in hippocampal histone acetylation and behavioral testing in isoflurane-treated mice.

## Methods

### Animals

All animal experiments were approved by the Animal Ethics Committee of Xiangya Hospital, Central South University, China (Approval number: 2011–11028). A total of 234 male C57BL/6 mice purchased from the Experimental Animal Center of Central South University were used for this study. Mice were housed in group cages(5–6 animals per cage) with free access to food and water. The environment was controlled on a 12/12-h light/dark cycle at a temperature of 25±2°C.

### Gas anesthesia and drug administration

The neonatal mice were exposed to 0.75% isoflurane three times (postnatal days 7, 8, and 9) in groups of 12–20 using a gas-delivery chamber. Each isoflurane exposure lasted 4 h. The gas was carried by 30% O_2_, and the total flow was controlled at 2 L/min. The concentration of isoflurane was measured in the gas-delivery chamber outlet using a Capnomac Ultima anesthesia monitor (Daetex-Ohmeda of GE Healthcare, Wauwatosa, WI, USA). The control group was exposed to 30% O_2_-enriched air. The environmental temperature of gas-delivery chamber was controlled at 36±1°C. Arterial blood specimens were obtained with an interval of 2 h during the first isoflurane exposure and immediately following the second and third exposures; mice were sacrificed by cervical dislocation and the hearts were quickly exposed, then the samples of blood in left cardiac ventricle were drew into syringe for blood-gas and blood glucose analyses.

To assess the effect of TSA on CFC memory in isoflurane-treated mice, TSA (Sigma-Aldrich, St. Louis, MO, USA) was dissolved in 4% dimethyl sulfoxide (DMSO), and the concentration was controlled at 0.5 μg/μl. TSA (2 mg kg^-1^) or vehicle was intraperitoneally injected 2 h before CFC training. An equal number of air-exposed mice were used as controls and also received TSA or vehicle.

### Behavioral testing

months after gas exposure, the mice underwent Open-field test and CFC trial. The mice were handled for 5 d, and on the day of behavioral testing they were transported to the laboratory at least 2 h before behavioral testing.

#### Open-field Test

Open-field test was performed in a white plastic box(75×75×45 cm).The mice were placed in the center of the box and allowed to explore it for 5 min under a weak light condition(about 5 lux), the travel trace was captured by a camera using software Smart JUNIOR(Panlab Harvard Apparatus, Barcelona, Spain). Locomotor activity of mice was measured by the total distance (centimeters) traveled in 5 min and anxiety level was assessed by the exploration time in the center of the open field.

#### CFC Trial

For CFC, the mice were placed in a pellucid Perspex chamber (40×30×26 cm) in a soundproof cabinet (75×60×45 cm). The training procedure of each mouse was recorded by a high-resolution camera located on the ceiling of the soundproof cabinet equipped with ANY-maze software (Stoelting Co, Wood Dale, IL, USA). The floor of the training chamber consisted of 28 iron bars that delivered electric footshocks. The CFC training was conducted for 5 min. At the beginning of the fifth minute, mice received a 0.75-mA footshock for 2 s. ANY-maze software was used to analyze the video files to determine whether or not the mice were in freezing behavior. The observation time-window for freezing behavior during CFC training was from the second minute until footshock administration. The CFC testing took place 24 h later, and we measured the freezing time in 3 consecutive min when the mice were placed into the same chamber.

### Tissue extraction and immunoblot analysis

At each time point after CFC training, mice were sacrificed by cervical dislocation. Each brain was quickly dissected and cut into coronal slices, and the cornu ammonis (CA)1 regions of the hippocampi were separated from transverse hippocampal slices under a dissecting microscope and were stored in liquid nitrogen. The details of micro-dissection and subsequent histone extraction and protein sample preparation were described in our previous study [14]. An equal amount of protein from each sample was separated by sodium dodecyl sulphate-polyacrylamide gel electrophoresis (SDS-PAGE) and then transferred onto polyvinylidene difluoride membranes. After blocking with 5% skim milk for 60 min, membranes were incubated overnight at 4°C with the following primary antibodies: anti-acetyl histone H3K9 (#9671, 1:1000; Cell Signaling Technology, Danvers, MA, USA), anti-acetyl histone H3K14 (#4318, 1:1000, Cell Signaling Technology), anti-acetyl histone H4K5 (#9672, 1:1000, Cell Signaling Technology), anti-acetyl histone H4K12 (SC-34266, 1:200; Santa Cruz Biotechnology, Santa Cruz, CA, USA), anti-histone H3 (ab1791, 1:3000, Abcam, Cambridge, UK), or anti-histone H4 (#2935, 1:1000; Cell Signaling Technology). Membranes were subsequently incubated with the secondary antibody (1:3000, Proteintech, Chicago, IL, USA) for 1 h at room temperature. Bands were developed with SuperECL Plus reagents (Thermo Fisher Scientific, Waltham, MA, USA). ImageJ 1.48 software (National Institutes of Health, Bethesda, MD, USA) was used to measure the relative acetyl-histone band densities.

### Tissue preparation and immunohistochemistry

Tissue preparation and immunohistochemistry procedures were performed as we previously described [14] with minor changes. Coronary brain sections were obtained at the same bregma range(-1.6 mm to bregma) from each group(n = 6) and 15-μm-thick slices were used for immunohistochemistry. Five slices from each brain were observed for assessing the c-Fos expression in the CA1 area. Processed coronal brain sections were successively incubated with primary antibody (12 h, 4°C), serum (10 min, room temperature), biotin-conjugated secondary antibodies (30 min, 37°C), streptavidin-peroxidase complex (30 min, 37°C), 3, 3’-diaminobenzidine (2–10 min, room temperature), and hematoxylin (2–5 min, room temperature). The primary antibody was anti-c-Fos (#2250, 1:200; Cell Signaling Technology). Finally, all sections were dehydrated, washed, and fixed onto gelatin-coated slides (China National Medicines, Shanghai, China). Tissue sections were observed using a Leica DM5000B microscope (Leica Microsystems CMS GmBH, Wetzlar, Germany). We quantified c-Fos-positive cells at 400× using ImageJ 1.48 software to assess expression levels in the CA1 area.

### Statistical analysis

All statistical tests were performed with SPSS 13.0 software (SPSS, Chicago, IL, USA). Data are expressed as mean ± standard deviation (SD). Differences in blood gas analysis and blood glucose value were analyzed by Student’s *t* test (pairwise comparisons) and two-way analysis of variance (ANOVAs) with two factors time and group. Differences in behavioral testing were analyzed by Student’s *t* test (pairwise comparisons) or one-way ANOVAs. Immunoblot and immunohistochemistry data were analyzed using one-way ANOVAs with Student—Newman—Keuls or Dunnett's tests. *P*<0.05 was considered statistically significant in all cases.

## Results

### Blood glucose and blood gas analysis during isoflurane exposure

Prolonged isoflurane anesthesia may result in respiratory depression and pathoglycemia. A previous study demonstrated that 1.5% isoflurane exposure for 6 h induced hypercapnia, hypoglycemia, and increased mortality in neonatal mice[15]. Therefore, we performed blood gas analysis and measured blood glucose during gas exposure. As shown in Table 1, there were no significant differences in blood pH, arterial oxygen tension, arterial carbon dioxide tension, and blood glucose between the two groups (*t* test, all *p*>0.05; two-way ANOVA, tests of between-subjects effects for group factor, all *p*>0.05, tests of between-subjects effects for time factor*group factor, all *p*>0.05). These data demonstrate that the exposure to 0.75% isoflurane for 4 h did not have any detrimental effect on respiratory function or blood glucose values in neonatal mice. In the current study, there was no mortality during gas exposure both 0.75% isoflurane or 30% O_2_-enriched air. These results suggest that the neurocognitive impairment following gas exposure was unlikely due to hypoxia/hypoventilation or pathoglycemia.

**Table 1:** Blood Gas and Blood Glucose Analyses during Gas Exposure.

Group	Time point	n	pH	PaO_2_, mmHg	PaCO_2_,mmHg	Hct, %	Glucose,mg/dl
30% O_2_-enriched air	2 h in 1st exposure	8	7.34±0.05	154.0±10.2	30.2±5.6	34.2±3.8	75.6±10.3
	4 h in 1st exposure	8	7.32±0.08	142.5±11.1	31.5±5.9	34.0±3.5	70.4±12.3
	2nd exposure	8	7.31±0.08	159.7±9.7	33.4±6.1	34.6±4.1	69.8±10.9
	3rd exposure	8	7.33±0.09	164.7±13.9	29.6±6.9	34.4±4.2	71.6±11.5
0.75% isoflurane	2 h in 1st exposure	8	7.37±0.07	164.2±9.6	33.4±5.8	34.1±4.1	70.4±12.7
	4 h in 1st exposure	8	7.38±0.06	155.7±13.6	35.6±6.8	34.7±4.0	55.5±13.2
	2nd exposure	8	7.40±0.07	153.8±15.6	34.8±5.4	34.3±3.7	54.6±13.7
	3rd exposure	8	7.37±0.06	160.1±12.3	33.6±6.8	34.5±3.5	60.5±11.5

Hct: hematocrit; PaCO_2_: arterial carbon dioxide tension; PaO_2_: arterial oxygen tension.

There were no significant differences in blood gases or blood glucose between mice exposed to isoflurane and those exposed to O_2_-enriched air. Mean (SD) values are shown.

### Neonatal exposure to isoflurane impaired neurocognitive function and induced histone acetylation dysregulation during CFC training in adult mice

**Fig 1:**
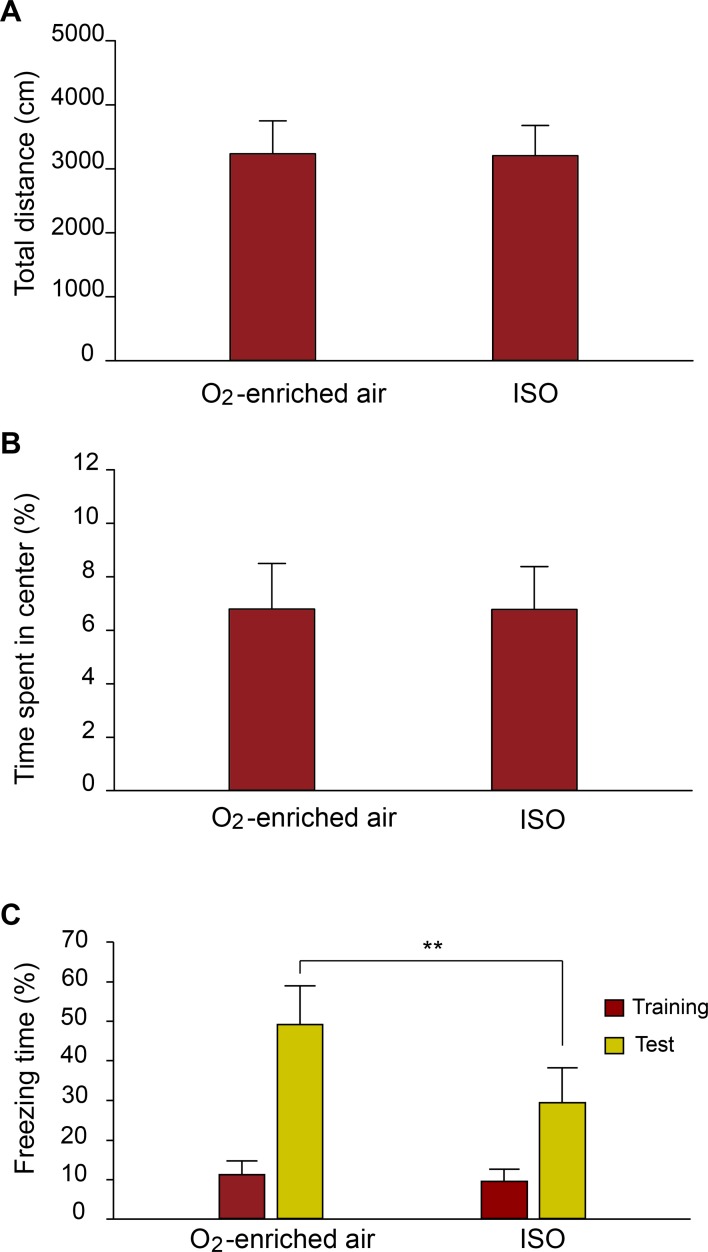
Effect of repeated neonatal isoflurane exposure on behavioral testing. (A) Mice that received repeated neonatal exposure to 0.75% isoflurane displayed normal traveled total distance in Open-field tests. (B) Mice that received repeated neonatal exposure to 0.75% isoflurane displayed normal affective state(anxiety level), which was measured by the percentage of time spent in the center of open-field. (C) Mice that received repeated neonatal exposure to 0.75% isoflurane displayed decreased freezing time (** p<0.01 versus control) during contexual fear conditioning (CFC) test. The control group inhaled 30% O_2_-enriched air during the neonatal period.

In the open-field test, there was no significant difference between O_2_-enriched air group and isoflurane group in the total distance and the percentage of time spent in the center of the open-field(t = 0.143, df = 22, *p* = 0.888; t = -0.027, df = 22, *p* = 0.979), which suggested repeated neonatal isoflurane exposure had no significant influence on locomotor activity and anxiety level (Fig 1A and 1B).

In the present study, we assessed the neurocognitive function of mice using CFC trials. During CFC training, all mice exhibited few freezing behavior before the footshock was given; there was no significant difference between O_2_-enriched air group and isoflurane group (t = 0.197, df = 22, *p* = 0.845; Fig 1C). During CFC testing, the isoflurane group showed a significant reduction in freezing time (t = 3.334, df = 22, *p* = 0.003), which suggested that their ability to forming fear-associated memory was impaired by isoflurane (Fig 1C).

**Fig 2:**
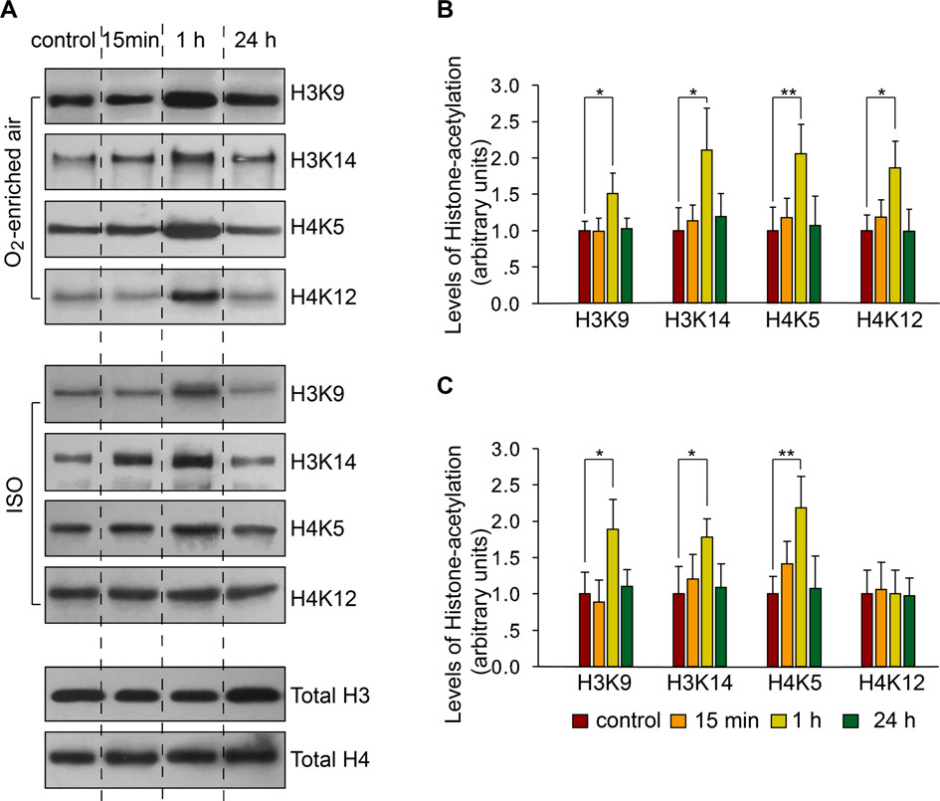
Repeated neonatal exposures to isoflurane induce H4K12 acetylation dysregulation in the CA1 hippocampal region in response to CFC training. (A) Representative images of western blots showing histone acetylation levels in the CA1 hippocampal region at 15 min,1 h, and 24 h after CFC training. Control mice were not subjected to CFC. (B) Quantification of the immunoblots from mice that received repeated exposure to 30% O_2_-enriched air. * p<0.05 and ** p<0.01 versus control. (C) Quantification of the immunoblots in mice that received repeated neonatal exposure to isoflurane. * p<0.05 and ** p<0.01 versus control. Mean (SD) values are shown.

We assessed the levels of histone acetylation in the CA1 hippocampal area at different time points after CFC training. The results showed that acetylation of H3K9, H3K14, H4K5, and H4K12 at 1 h after CFC training were significantly increased relative to baseline level at naive condition in the mice exposed to 30% O_2_-enriched air (H3K9 F_(3,20)_ = 10.358, *p*<0.001; H3K14 F_(3,20)_ = 10.728, *p*<0.001; H4K5 F_(3,20)_ = 14.288, *p*<0.001; H4K12 F_(3,20)_ = 12.494, *p*<0.001; Multiple comparisons H3K9_1 h VS control_, *p* = 0.023; H3K14_1 h VS control_, *p* = 0.036; H4K5_1 h VS control_, *p* = 0.009; H4K12_1 h VS control_, *p* = 0.023), but there were no differences among baseline, 15 min, and 24 h after CFC training (all *p*>0.05 in multiple comparisons, Fig 2B). In the isoflurane-exposed mice, H3K9, H3K14, and H4K5 acetylation were similarly increased 1 h after CFC training (H3K9 F_(3,20)_ = 12.322, *p*<0.001; H3K14 F_(3,20)_ = 6.841, *p* = 0.002; H4K5 F_(3,20)_ = 13.006, *p*<0.001; Multiple comparisons H3K9_1 h VS control_, *p* = 0.011; H3K14_1 h VS control_, *p* = 0.014; H4K5_1 h VS control_, *p* = 0.002), while H4K12 acetylation was unaffected (F_(3,20)_ = 0.070, *p* = 0.975; Fig 2C).

### TSA ameliorates isoflurane-induced neurocognitive impairment by manipulating hippocampal histone acetylation

**Fig 3:**
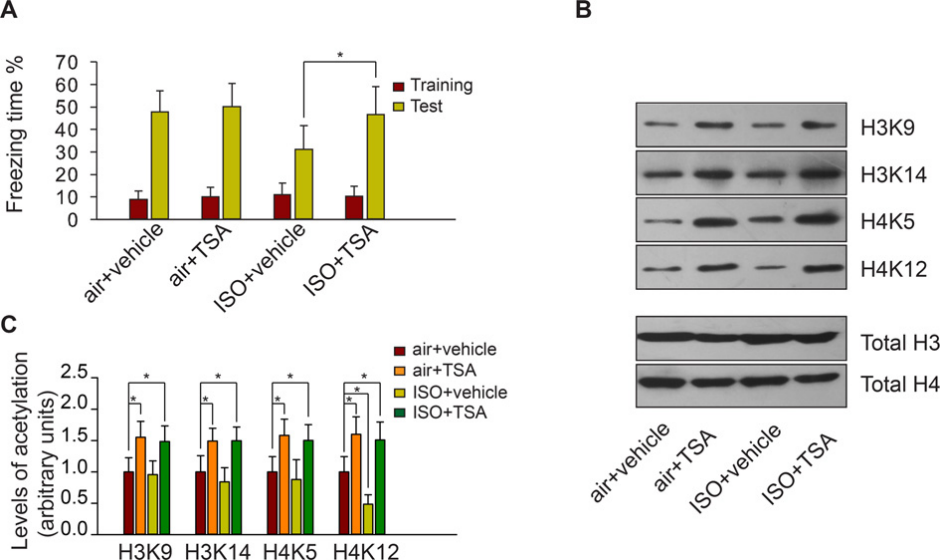
TSA injection improved CFC performance and hippocampal histone acetylation in mice that received repeated neonatal exposures to isoflurane. (A) TSA injection improved CFC performance in mice that received repeated neonatal exposures to isoflurane. The freezing times of each group (n = 12 mice per group) during CFC training and testing are shown. * p<0.05. (B) Representative images of western blots showing histone acetylation levels in the CA1 hippocampal region for each group 1 h after CFC training. (C) Quantification of (B). * p<0.05 versus the Air+vehicle group. Mean (SD) values are shown.

To examine whether the HDACi TSA was capable of attenuating isoflurane-induced neurocognitive impairment, we divided the mice into four groups: an air+vehicle group subjected to three neonatal exposures to O_2_-enriched air and injected with DMSO (vehicle) 2 h before CFC training, an air+TSA group with neonatal exposure to O_2_-enriched air injected with TSA, an ISO+vehicle group that received repeated neonatal exposures to 0.75% isoflurane and vehicle injection 2 h before CFC training, and an ISO+TSA group that received isoflurane exposures and TSA injection. During the CFC training phase, there were no differences in freezing times among the four groups (F_(3,44)_ = 0.606, *p* = 0.615; Fig 3A). When re-exposed to the CFC chamber 24 hours later, the ISO+vehicle group exhibited a significant reduction in freezing behavior compared with the other three groups (F_(3,44)_ = 6.545, *p* = 0.001; Fig 3A). TSA only facilitated memory formation in mice with neonatal isoflurane exposure(Multiple comparisons: ISO+vehicle VS ISO+TSA, *p* = 0.037; air+vehicle VS air+TSA, *p* = 0.992) (Fig 3A).

At 1 h after CFC training, western blotting analysis showed that TSA increased H3K9, H3K14, H4K5, and H4K12 acetylation in the hippocampal CA1 area in all groups (all *p*<0.05 in multiple comparisons). H4K12 acetylation in the ISO+vehicle group was significantly decreased compared with the air+vehicle group (F_(3,20)_ = 26.392, *p*<0.001; ISO+vehicle VS air+vehicle in multiple comparisons, *p* = 0.012; Fig 3C).

### TSA injection increased c-Fos positive hippocampal neurons after CFC training

**Fig 4:**
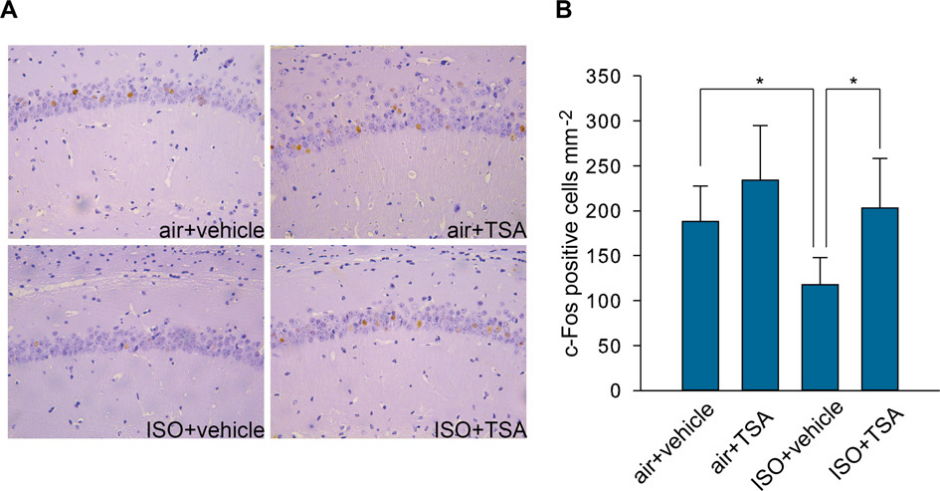
TSA injection improved c-Fos expression in response to CFC training in mice that received repeated neonatal exposures to isoflurane. (A) Representative immunohistochemistry images of c-Fos in the CA1 hippocampal region 1 h after CFC training. Hippocampal neuronal nuclei were stained purple with hematoxylin, and nuclear c-Fos protein is stained brown by DAB. (B) c-Fos-positive cell counts. Mice that received repeated neonatal exposures to isoflurane showed reduced numbers of c-Fos-positive neurons in the CA1 hippocampal region 1 h after CFC training, but this decrease was rescued by systemic TSA administration. * p<0.05. Mean (SD) values are shown.

c-Fos belongs to the activator protein-1 family of transcription factors and is an immediate early gene (IEG). Many previous studies have demonstrated that c-Fos expression is rapidly elevated by various experiential stimuli including conditioned and unconditioned aversive stimuli [16], sensory stimuli (e.g., auditory, visual, tactile, olfactory)[17–20], and various learning task [21–29]. Assessing c-Fos provides information about synaptic plasticity and neuronal activation required for long-term memory formation [27, 30]. In our study, compared with the air+vehicle group, the ISO+vehicle group exhibited a reduction in the number of c-Fos-positive cells in the CA1 area (F_(3,20)_ = 6.301, *p* = 0.003; ISO+vehicle VS air+vehicle in multiple comparisons, *p* = 0.047; Fig 4B). TSA increased the number of c-Fos-positive cells in CA1 1 h after CFC training in mice with repeated neonatal exposure to isoflurane (ISO+vehicle VS ISO+TSA in multiple comparisons, *p* = 0.044, Fig 4B).

## Discussion

The mechanism underlying neurocognitive impairment induced by inhalational anesthetic in the developing brain is thought to involve neurodegeneration, although the involved pathways are not entirely clear. The anesthetic properties of inhalational anesthetic are ascribed to a compound effect on the potentiation of γ-aminobutyric acid (GABA_A_)-gated receptors and inhibition of N-methyl-D-aspartate (NMDA)-gated receptors [31]. Several previous studies indicated that the ability of inhalational anesthetic to induce neurodegeneration in the developing brain is due to its effects on these two receptor types [32–34]. We performed repeated exposure to 0.75% isoflurane in neonatal mice and found that this mode of exposure can cause significant neurocognitive impairments without any changes in locomotor activity and anxiety levels in adult animals. Treatment with 0.75% isoflurane approximates an 0.5 minimum alveolar concentration (MAC) in adult mice, and the MAC in neonatal mice for isoflurane is higher than for adults [5]. In contrast to other studies describing that a single exposure with approximately 1 MAC concentration or prolonged exposure in the developing brain led to neurocognitive impairment, we found that the repeated neonatal exposure to a lower isoflurane dose could also induce neurocognitive impairment.

We used CFC testing to assess mouse neurocognitive function; this paradigm is commonly used to assess hippocampus-dependent associative learning and memory, and previous findings have demonstrated that the acetylation of several specific histone lysine residues (H3K9\14 and H4K5\8\12\16) in the hippocampus might be related to CFC memory formation[6, 35, 36]. We observed alterations in H3K9, H3K14, H4K5, and H4K12 acetylation in the CA1 hippocampal region at different time points after CFC training. Here, we choose to focus on the specific hippocampal subregion CA1 instead of whole hippocampus because CA1 is the major subreigion that has substantial reciprocal anatomical connections with the amygdala, which maybe hold significance for formation of CFC memory[37, 38],and several functional neuronal imaging studies demonstrated learning-related alterations occurred mainly in CA1 region instead of other subregions[39–41]. The results showed that the acetylation levels of these four histone residues were increased in the O_2_-enriched air exposure group 1 h after CFC training and returned to baseline levels within 24 h. In contrast to the O_2_-enriched air group, CFC training did not enhance H4K12 acetylation in the CA1 area of mice in the isoflurane exposure group. These data suggest that neurocognitive dysfunction induced by repeated exposure to isoflurane in neonatal mice was likely related to the dysregulation of hippocampal H4K12 acetylation. Histone acetylation is one of the mechanisms underlying local and global control of chromatin structure. A number of studies have reported that chromatin remodeling via histone acetylation responds quickly to various experiential stimuli to regulate downstream alterations in cellular gene expression [42–44]. Therefore, the dysregulation of histone acetylation following a learning task could result in deficiencies in memory-related gene expression and subsequent learning and memory dysfunction. In addition, it should be noted that the dysregulation of hippocampal histone acetylation only affected the specific lysine residue H4K12 in mice with neonatal isoflurane exposure. Peleg et al. [6] reported that impaired H4K12 acetylation also plays a role in age-related neurocognitive impairment because it leads to decreased expression of an array of genes underlying memory formation. It seems the neuropathologic basis for inhalational anesthetic-induced neurocognitive impairment may overlap with that for the cognitive dysfunction in the aged brain, although the shared underlying mechanisms remain unknown. Moreover, several previous studies[45–47] indicated that the acetylation of H3K9/14 and H4K5 also contributed greatly to learning, memory, and synaptic plasticity, and among them, the dysregulation of H3K9 acetylation has been reported to play a particularly important role in neurocognitive dysfunction related to iron overload[7]. However, no changes in the acetylation of these lysine residues were detected in animals with memory impairment induced by neonatal isoflurane exposure.

In previous studies, administration of global HDACis such as TSA or sodium butyrate was found to increase long-term potentiation (LTP) at Schaffer collaterals in CA1 of the hippocampus and rescue memory deficits in both aged and gene-mutant mice [6, 8–11]. Here, we found that TSA enhanced CFC memory formation in mice that underwent repeated neonatal exposure to isoflurane and exhibited memory impairment in adulthood but not in control mice. Similarly, several previous reports [8, 10, 48] also demonstrated that memory deficits in some genetic mouse models were mitigated by HDACi treatment, whereas normal cognitive function in wild-type mice was unaffected. Moreover, the negligible effect of TSA on CFC trials in the control group might be explained by the ‘‘ceiling effect” of animals’ fear response induced by an intense noxious stimulation. For instance, Itzhak et al. [48] reported that the maximum freezing response (“ceiling”) in mice during CFC trials was reached when the footshock current intensity reached 0.35 mA. It is possible that freezing responses are weakened and decrease to a lower level than the ceiling when the current intensity of footshock is less than 0.35 mA, in which case an effect of TSA on control mice with normal neurocognitive function could be detected. TSA is able to inhibit Class I (nuclear) and II (nuclear and cytoplasmic) HDACs both in vivo and in vitro, resulting in enhanced histone acetylation and subsequent changes in the expression of specific genes[49, 50]. We administered TSA (2 mg/kg) by intraperitoneal injection 2 h before CFC training, which was based on a previous study[8], and found that H3 and H4 acetylation in the CA1 hippocampal region markedly increased 1 h after CFC training. Notably, this model of TSA administration ameliorated the dysregulation of H4K12 acetylation following CFC training in mice with neonatal isoflurane exposure. We also observed changes of c-Fos expression 1 h after CFC training with or without TSA injection. c-Fos is considered a marker of synaptic plasticity and neuronal activation and is used to identify activated neurons for neuronal pathway tracing[51]. Our results showed that c-Fos expression was lower 1 h after CFC training in mice that received repeated neonatal isoflurane exposure, and the systemic TSA was able to rescue this gene expression deficit following the CFC task. These observations were in line with the observed effect on H4K12 acetylation.

There is a wide range of mechanisms that contribute to the protective effect of HDACis on neurocognitive function, including epigenetic and non-epigenetic pathways [52, 53]. Most studies have focused on their blocking effects on HDACs, which increase the acetylation levels of histone and facilitated downstream gene products. In addition, several recent studies showed HDAC inhibitors improved the memory function also through the activation of specific genes mediated by the cAMP response element-binding protein (CREB)-CREB binding protein (CBP) transcriptional complex [36, 54, 55]. However, HDACis can also promote the acetylation of some non-histone substances that participate in learning and memory-related cellular and molecular processes. For instance, Green and coworkers[56] reported that nicotinamide’s inhibitory effect on class III NAD(+)-dependent HDACs rescued cognitive deficits in a transgenic mouse model of AD through a mechanism involving the increased acetylation alpha-tubulin and MAP2c and reduced Thr231-phospho-tau. Regardless of how many mechanisms remain to be identified, HDACis have shown greater therapeutic potential for cognitive defects caused by a variety of factors[6, 8–11]. Our results are consistent with the previous studies described above and provide new evidence supporting the hypothesis that HDACis may be useful for treating neurocognitive impairment induced by inhalational anesthetics.

There are several noteworthy limitations of this study: First, it should be noted that the behavioral testing paradigm used in this study to assess learning and memory did not distinguish the different memory processes, including acquisition, consolidation and retrieval. Therefore, there is a possibility that the memory defect induced by neonatal isoflurane exposure may be just derived from the impairment of a specific memory process, such as acquisition impairment. Second, our observations just focused on the hippocampal CA1 subregion after mice received repeated neonatal exposure and intraperitoneal injection. Although histone acetylation changes in CA1 region were likely to play an important role in contributing to the observed effects on behavioral testing, it cannot be excluded that the similar changes in other regions of brain were implicated as well. Third, because we did not assess acetylation in other lysine sites or perform a genome-wide analysis of transcription after CFC, it is possible that the dysregulation of histone acetylation for other specific sites or expression deficits in other memory-related genes were overlooked by us. At last, we also did not assess the interaction between histone acetylation and target gene promoters using chromatin immunoprecipitation, and therefore further studies are required to identify specific effect of H4K12 acetylation on transcription regulation of genes in mice with memory impairment induced by neonatal isoflurane exposure together with the assessment of other memory-related genes.

In conclusion, our findings suggest that memory impairment induced by repeated neonatal exposures to 0.75% isoflurane is associated with dysregulated acetylation of histone H4K12 in the hippocampal CA1 region, which probably affects the downstream expression of memory-related genes such as c-Fos. TSA mitigated the isoflurane-induced memory impairment, most likely by enhancing histone acetylation levels and increasing c-Fos gene expression in the hippocampus.
